# Organization and variation of the Tetraonidae (Aves: Galliformes) mitochondrial DNA control region

**DOI:** 10.1080/23802359.2017.1361345

**Published:** 2017-08-23

**Authors:** Zuhao Huang, Dianhua Ke

**Affiliations:** School of Life Sciences, Jinggangshan University, Ji’an, Jiangxi, China

**Keywords:** Mitochondrial DNA, control region, structure, variation, Tetraonidae

## Abstract

The mitochondrial DNA control region is the most polymorphic region of the mitochondrial genome. To infer the organization and variation of Tetraonidae mitochondrial DNA control region, the entire control region sequences of 18 species were analyzed. The length of the control region sequences ranged from 1127 bp (*Bonasa sewerzowi*) to 1156 bp (*Centrocercus minimus*). The average genetic distances among the species within the genera varied from 0.53% (*Tympanuchus*) to 9.42% (*Bonasa*). The average genetic distances showed insignificantly negative correlation with ts/tv. Five conserved sequence boxes in the domain II of Tetraonidae sequences were identified. The alignment of the Tetraonidae five boxes and CSB-1 sequences showed a few sequence variations. The results indicated that the genus *Dendragapus* might not be monophyletic.

Tetraonidae is a group of birds from the order Galliformes, including six genera, eighteen species. Lucchini et al. (2001) preliminary described the structure of the mtDNA control region of Tetraonidae. But the rates and patterns of molecular evolution of Tetraonidae control region are not known. In the present study, we examined the organization and variation of the control region of Tetraonidae species retrieved from GenBank (Table 1). The aim is to characterize the structural features and patterns of sequence evolution of the Tetraonidae mitochondrial DNA control region.

**Table 1:** Species examined and source of sequence data in the present study.

Genus	Species	Length of the control region	Accession Number	Sources
	*Dendragapus falcipennis*	1149	AJ297164	Lucchini et al. 2001
*Dendragapus*	*Dendragapus canadensis*	1147	AJ297163	Lucchini et al. 2001
	*Dendragapus obscurus*	1150	AJ297161	Lucchini et al. 2001
	*Lagopus lagopus*	1140	AJ297169	Lucchini et al. 2001
*Lagopus*	*Lagopus muta*	1142	AJ297170	Lucchini et al. 2001
	*Lagopus leucura*	1143	AJ297168	Lucchini et al. 2001
	*Tetrao tetrix*	1147	NC024554	Li et al. 2016
	*Tetrao mlokosiewiczi*	1145	AJ297173	Lucchini et al. 2001
*Tetrao*	*Tetrao urogallus*	1146	DQ323553	Alda et al. 2016, unp
	*Tetrao parvirostris*	1143	AJ297178	Lucchini et al. 2001
	*Bonasa bonasia*	1141	NC020591	Shen et al. 2010
*Bonasa*	*Bonasa sewerzowi*	1127	NC025318	Li et al. 2014
	*Bonasa umbellus*	1146	AJ297157	Lucchini et al. 2001
	*Centrocercus urophasianus*	1151	GQ902785	Oyler-McCance et al. 2010, unp
*Centrocercus*	*Centrocercus minimus*	1156	GQ902778	Oyler-McCance et al. 2010, unp
	*Tympanuchus phasianellus*	1148	AJ297176	Lucchini et al. 2001
*Tympanuchus*	*Tympanuchus cupido*	1148	AJ297171	Lucchini et al. 2001
	*Tympanuchus pallidicinctus*	1147	AJ297174	Lucchini et al. 2001
	*Bambusicola thoracica*	1146	EU165706	Shen et al. 2010
Outgroup	*Bambusicola fytchii*	1174	NC020583	Shen et al. 2010

A total of 18 species from 6 genera belonging to the Tetraonidae family were analyzed. All the Tetraonidae species had only one control region. The control region spans the region between the genes for tRNA^Glu^ and tRNA^Phe^ in the Tetraonidae species. The length of the control region is relatively conserved, about 1145 ± 11 bp, but that of *Bonasa sewerzowi* is only 1127 bp.

The average nucleotide composition of Tetraonidae control region sequences was as follows: 25.92%A, 33.65%T, 14.01%G and 26.43%C, with a bias against G. The amount of A + T was more than that of G + C among whole control region, especially in domain III, same as reported other avian control region (e.g. Marshall and Baker 1997; Ruokonen and Kvist 2002; Huang and Ke 2016).

Genetic distances between species ranged from 0.27% (between *Tympanuchus phasianellus* and *Tympanuchus cupido*) to 13.05% (between *Tetrao urogallus* and *Bonasa sewerzowi*), showing a wide range of divergences. The average genetic distances among the species within the genera varied from 0.53% (*Tympanuchus*) to 9.42% (*Bonasa*). The average genetic distances showed insignificantly negative correlation with ts/tv (*r* = −0.6852, *p* > .05).

Nucleotide substitutions occur more frequently in peripheral domains. Average substitution rate for the three domains was 0.51:0.22:0.27, corresponding to relative proportions of 5:2:3, respectively. Among all the genera of Tetraonidae, domain I is the most variable of the three domains (Figure 1).

**Figure 1.**
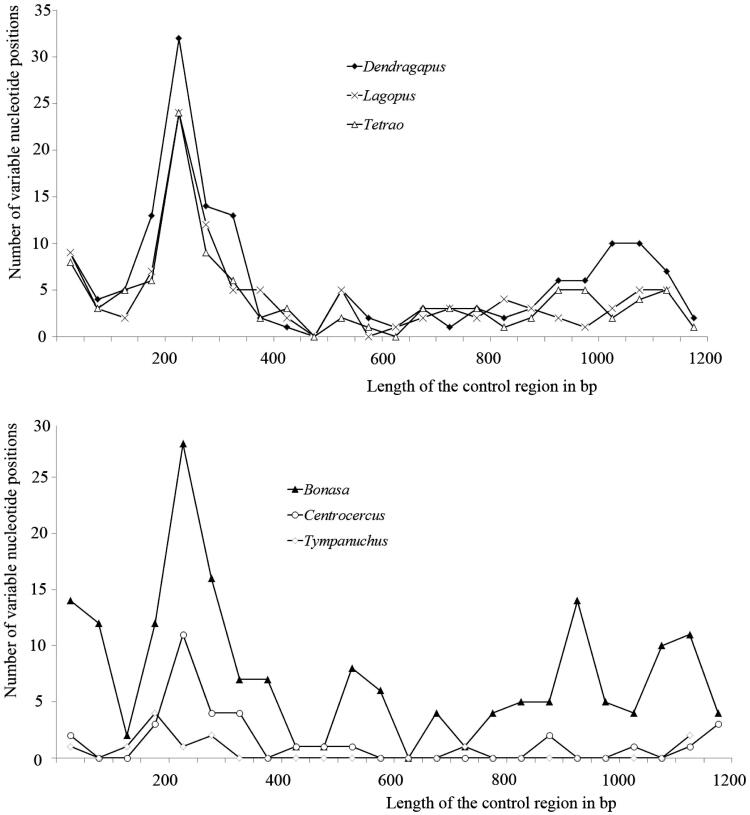
Distribution of the variable sites in the control region. The number of variable sites within genera has been plotted in 50-bp intervals.

Five conserved sequence boxes in the domain II of Tetraonidae sequences were localized and identified as boxes F, E, D, C and B. These foxes were also identified by Randi and Lucchini (1998) in *Alectoris* sequences, except B-box. In F-box, 21 of 28 nucleotide positions were fully conserved among the Tetraonidae sequences. There were both four nucleotide positions were variable in C-box and B-box, while there were only one and two nucleotide positions were variable in D-box and E-box, respectively.

On the basis of hierarchical likelihood ratio tests (hLRTs) as implemented in ModelTest 3.0, the model general time reversible (GTR) model + gamma distribution (G) +invariable (I) was used (GTR + G +I, −lnL = 4643.26, AIC = 9372.72, BIC = 9711.70). Maximum likelihood method was used to reconstruct the phylogenetic trees based on GTR + G +I model. Member of Tetraonidae was formed an alone clade, as a sister to out-group (Figure 2). Species of *Bonasa* was the first to split from the Tetraonidae lineage. All the genera could be discriminated by their distinct clades in the phylogenetic tree except *Dendragapus*. Our results indicated that the genus *Dendragapus* might not be monophyletic.

**Figure 2.**
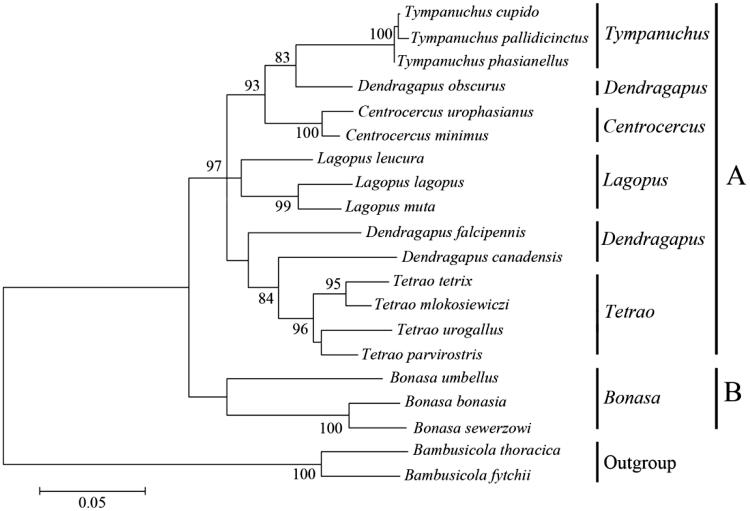
Phylogenetic tree of Tetraonidae constructed from mitochondrial DNA control region sequence. Numbers at nodes indicate bootstrap values (≥80%) from 1000 replications.
